# Comparation of predictive value of CAT and change in CAT in the short term for future exacerbation of chronic obstructive pulmonary disease

**DOI:** 10.1080/07853890.2022.2055134

**Published:** 2022-03-26

**Authors:** Ling Lin, Qing Song, Wei Cheng, Cong Liu, Yi-Yang Zhao, Jia-Xi Duan, Jing Li, Dan Liu, Xin Li, Yan Chen, Shan Cai, Ping Chen

**Affiliations:** a Department of Respiratory and Critical Care Medicine, the Second Xiangya Hospital, Central South University, Changsha, Hunan, China; b Research Unit of Respiratory Disease, Central South University, Changsha, Hunan, China; c Diagnosis and Treatment Center of Respiratory Disease, Central South University, Changsha, Hunan, China; d Department of Respiratory, The Eighth Hospital, Changsha, Hunan, China in; e Division 4 of Occupational Diseases, Hunan Prevention and Treatment Institute for Occupational Diseases, Changsha, China

**Keywords:** COPD, change in CAT, baseline CAT score, exacerbation

## Abstract

**Purpose:**

Our study aimed to compare the predictive value of the COPD Assessment Test (CAT) score at baseline and short-term change in CAT for future exacerbations in chronic obstructive pulmonary disease (COPD) patients.

**Methods:**

This was a multicentre prospective study. Patients with COPD were recruited into the study and followed up for one year. CAT score and exacerbation in the previous year were collected at baseline. Change in CAT was defined as CAT score changing between baseline and the 6-month follow-up. Exacerbation was recorded during the one-year follow-up from 0^th^ to 12^th^ month.

**Result:**

A total of 536 patients were enrolled for final analysis. The mean baseline CAT score was 14.5 ± 6.6 and the median (IQR) change in CAT was −2 (8). On Cox regression analysis, baseline CAT score, change in CAT and history of exacerbation were independent risk factors for exacerbation in the one-year follow-up. Compared with the *r* value of correlation between baseline CAT score and frequency of exacerbations during the one-year follow-up (*r* = 0.286, *p* < .001), that correlation between the change in CAT and frequency of exacerbations during follow-up was higher (*r* = 0.421, *p* < .001). The receiver operating characteristic (ROC) curves showed that change in CAT had a better predictive capacity for future exacerbation than baseline CAT (0.789 versus 0.609, *p* = .001). The ROC showed that change in CAT also had a better predictive capacity for future exacerbation than exacerbation in the previous year (0.789 versus 0.689, *p* = .011).

**Conclusion:**

The correlation between baseline CAT score and future exacerbation was weak, however, the correlation between change in CAT and future exacerbation was moderate. Change in CAT in the short term had a better predictive value for future exacerbations of COPD than baseline CAT and exacerbation in the previous year.

## Introduction

Chronic obstructive pulmonary disease (COPD) is the third leading cause of death worldwide [1]. Exacerbations are the key outcome of COPD patients, defined as exacerbations of respiratory symptoms leading to additional treatment [2]. Exacerbation of COPD will accelerate hospitalisation rates [3], increase mortality [4], reduce the quality of life [5,6]. Therefore, prevention of exacerbations is one of the important measures of COPD management strategies.

At present, there are many methods for predicting future exacerbations. Among them, the history of exacerbation is one of the most powerful predictors [7,8]. It is usually applied in clinical settings, but the disadvantage is that it is more subjective, leading to a higher missed diagnosis rate. Relevant studies have confirmed that the poor quality of life of COPD patients is associated with future exacerbations [6]. The Global Initiative for Chronic Obstructive Lung Disease (GOLD) recommends the use of the COPD Assessment Test (CAT), which evaluates and quantifies the impact of COPD symptoms on the health of patients [9]. The CAT score observed at a point in time is a reliable indicator for predicting future exacerbations. Recent studies have shown that the higher the CAT score, the higher the risk of future exacerbations [10]. Jo et al. [11] indicated that a baseline CAT ≥15 can predict future exacerbations. However, the disease trajectory is also an important basis for predicting the risk of exacerbation. Recent research based on analysis of TORCH (Towards a Revolution in COPD Health) trial data, found that clinically important deterioration (CID) occurred within 6 months, including moderate to severe deterioration, a decrease of forced expiratory volume in 1 s (FEV1) or the deterioration of the total score of St George's Respiratory Questionnaire (SGRQ), is associated with an increased risk of long-term deterioration [12]. SGRQ is another questionnaire used to assess the health status of people with respiratory disease [13]. At the individual level, the investigator determined −2 units to be the minimal clinically important difference (MCID) of the change in CAT score, with an SGRQ change of −4 as an anchor [14]. Zhao et al. [15] found that the CID based on 2 points of CAT deterioration (CID-C) shows a good predictive value for the risk of exacerbation in the future. Therefore, short-term changes in CAT scores may be associated with the risk of exacerbation. However, there is no specific study to clarify the predictive value of changes in CAT for future exacerbations alone. Additionally, there is no research comparing the predictive value of baseline CAT score and change in CAT for future exacerbations. Therefore, the purpose of this study is to compare the predictive value of baseline CAT score and short-term change in CAT for future exacerbations of COPD.

## Methods

### Study design and subjects

This was a multicentre, prospective and observational study. All subjects were from the outpatient COPD database of the Second Xiangya Hospital of Central South University (ChiCTR-POC-17010431) from December 2016 to October 2020. According to GOLD 2017 guidelines, COPD was diagnosed when a ratio of FEV1 to forced vital capacity (FEV1/FVC) < 0.70 after inhaling a bronchodilator [16]. We excluded patients with a history of bronchiectasis, asthma, lung cancer or pneumonia, or severe heart, liver or kidney disease. This study was conducted in accordance with the Declaration of Helsinki and approved by the Ethics Committee of the Second Xiangya Hospital of Central South University. All patients provided written informed consent.

### Data collection and definition

Clinical characteristics included age, sex, education degree, body mass index (BMI), smoking history, biofuel and occupational exposure history, CAT, Modified Medical Research Council Dyspnoea Scale (mMRC), Clinical COPD Questionnaire (CCQ), pulmonary function, inhalation therapy drugs and exacerbations (moderate to severe) in the previous year; these were collected at baseline. As for smoking history, we defined “smoker” as smoking exposure more than 10 pack-years, “ex-smoker” as not less than 10 pack-years, but smoking cessation more than 6 months, “never smoker” as smoking exposure under 10 pack-years [17]. COPD disease severity was classified using the GOLD guidelines and was divided into four stages: I (FEV1 ≥ 80% predicted), II (FEV1 50–80% predicted), III (FEV1 30–50% predicted), or IV (FEV1 < 30% predicted). The total score of CAT ranges from 0 to 40. At the 6-month visit, the results of the CAT score, medication possession ratio (MPR) and exacerbations during the 6 months were recorded. Adherence was calculated using MPR. MPR was calculated by summing the days of medication supply provided and dividing by the total time treated. Adherence was classified as follows: Poor adherence (MPR < 80% or MPR > 120%) and Adherence [18]. Then, data on exacerbation over one year were collected at the 12-month follow-up.

### Definition of baseline CAT score and change in CAT

Baseline CAT score was collected at baseline. Change in CAT was defined as CAT score changing between baseline and the 6-month follow-up.

### Definition of exacerbation

Moderate exacerbation was defined as exacerbation of respiratory symptoms requiring antibiotics and/or oral corticosteroids; severe exacerbation was defined as exacerbations requiring hospitalisation or emergency room admission for more than 2 days. If the patient had at least two exacerbations or died during the follow-up period, they were recorded as experiencing a frequent exacerbation.

### Statistical analysis

SPSS 26.0 (IBM, Armonk, NY, USA) was used for statistical analysis of the data. Continuous variables were expressed as mean ± standard deviation or median and interquartile range as appropriate. Continuous variables that were normally distributed and showed homogeneity of variance were tested using Student’s t-test; otherwise, non-parametric tests were used. The chi-square test was used for categorical variables. Association analysis was performed using Spearman’s rank correlation coefficient. A multivariate Cox regression analysis was performed for identifying factors predicting exacerbation during one-year follow-up, by including variables that were significant (*p* < .05) on univariate analysis. The multivariate stepwise logistic regression analysis was used to analyse the clinical features associated with the change in CAT. The ROC curve was calculated and the area under the curve (AUC) was compared using the *Z*-test. For all analyses, a *p*-value of <.05 was considered statistically significant.

## Results

### Baseline characteristics

**Figure 1.**
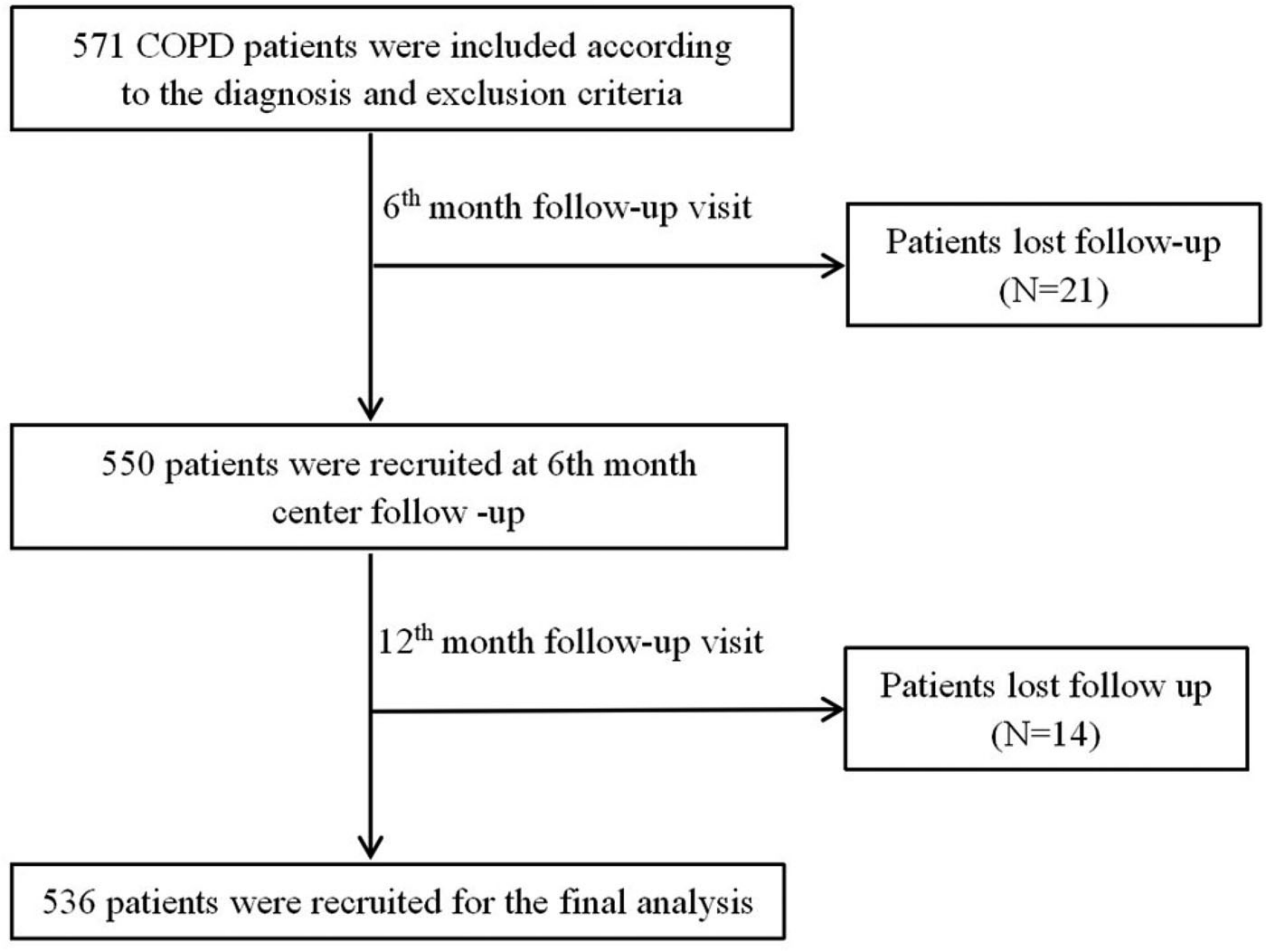
Flow diagram of the inclusion of study. Abbreviations: COPD, Chronic Obstructive Pulmonary Diseases.

A total of 571 patients with COPD were initially enrolled. At a 6-month visit, 21 patients were excluded from the study due to loss of contact. At the 12-month follow-up, 14 patients dropped out due to loss of contact. Finally, we recruited 536 patients for the final analysis (Figure 1). Baseline demographic and clinical characteristics are shown in Table 1. Overall, 89.0% of patients were male, and the mean age of all patients was 64.0 ± 8.0 years. Most patients had a history of smoking exposure, with 38.6% being current smokers and 42.9% ex-smokers. The mean CAT score was 14.5 ± 6.6 and the median (IQR) FEV1% was 50.3 (28.8). The proportion of patients who experienced moderate to severe exacerbations in the previous year was 58.0% and 33.8% experienced frequent exacerbations. The distribution of inhalation therapy was as follows: LAMA(30.5%), LAMA/LABA (7.0%), ICS/LABA (9.6%), ICS/LABA/LAMA (51.0%), Others (1.6%) including ICS/LAMA, short-acting bronchodilators and no inhalation medication.

**Table 1.** Baseline demographics and clinical characteristics of the patients.

Variables	Total group (*n* = 536)
Age (years)^a^	64.0 (8.4)
Sex^b^	
Male	477 (89.0)
Education^b^	
Primary school	207 (38.6)
Junior	205 (38.2)
High school	84 (15.7)
University	40 (7.5)
BMI (kg/m^2^)^a^	22.5 (3.2)
Smoking status	
Current-smoker^b^	207 (38.6)
Former smoker^b^	230 (42.9)
Never smoker^b^	99 (18.5)
Smoke index, pack-year^c^	35 (36)
Biofuel exposure^b^	169 (31)
Occupational exposure^b^	220 (41.1)
CAT^a^	14.5 (6.6)
0–10^b^	155 (28.9)
11–40^b^	381 (71.1)
mMRC^c^	2 (2)
CCQ^a^	18.9 (9.2)
FEV1^c^	1.2 (0.75)
FEV1 (% predicted)^c^	50.3 (28.8)
FEV1/FVC^c^	45.0 (20.3)
Exacerbationsin the past year^c^	1 (2)
Exacerbations in the past year^b^	
0	225 (42.0)
1	135 (25.2)
≥2	176 (33.8)
Gold grade^b^	
I	48 (9.3)
II	213 (40.0)
III	206 (38.7)
IV	69 (13.0)
Therapies^b^	
LAMA	164 (30.5)
ICS + LABA	52 (9.6)
LABA + LAMA	38 (7.0)
ICS + LABA + LAMA	274 (51.0)
Others	8 (1.6)

^a^Mean (SD); ^b^Counts with percentage are indicated; ^c^Median (IQR).

Abbreviations: BMI, Body Mass Index; COPD, Chronic Obstructive Pulmonary Diseas; CAT, COPD Assessment Test; CCQ, Clinical COPD Questionnaire; FEV1, Forced Expiratory Volume in one second; FVC, Forced Vital Capacity; GOLD, Global Initiative for Chronic Obstructive Lung Disease. ICS, inhaled corticosteroids; IQR, interquartile range; LABA, long-acting β-2-agonist; LAMA, long-acting muscarinic antagonist; mMRC, modified medical research council dyspnoea scale.

### Characteristics during one-year follow-up

The mean CAT at 6-month follow-up was 12.2 ± 6.6, with a median (IQR) change in CAT of −2 (8) from baseline to 6-month follow-up. Of the 536 patients, 63.6% were with adherence to therapy, 21.5% of patients had at least one moderate to severe exacerbation during the 6-month follow-up. The median (IQR) number of exacerbations in a 6-month visit was 0 (1) (Table 2). 36.0% had at least one moderate to severe exacerbation, 16.0% had frequent exacerbations, and 21.8% had severe exacerbations during 12-month follow-up (Table 3). The median (IQR) number of exacerbations during the 12-month follow-up among patients with exacerbations in the previous year and no exacerbations in the previous year were 0 (1) and 0 (0), respectively (*p* < .001). In addition, patients with exacerbations in the previous year were more likely to suffer future exacerbation than patients with no exacerbation in the previous year (Table S1).

**Table 2.** Clinical Characteristic of a patient in the 6-month follows up.

Variables	Total group (*n* = 536)
CAT at 6^th^ month， Mean（SD）	12.2（6.6）
Change of CAT	−2 (8)
Median (IQR)	
Exacerbations in the 6 months， Median（IQR)	0 (1)
Exacerbations in the 6 months, *n* (%)	
Yes	115 (21.5)
No	421 (78.5)
MPR, *n* (%)	
Adherence	341 (63.6)
Poor adherence	195 (37.4)

Abbreviations: CAT, COPD Assessment Test; Change in CAT, CAT score changing between baseline and the 6-month follow-up; MPR: medication possession ratio.

**Table 3.** Exacerbation of patient during the one-year follow-up.

Variables	Total group (*n* = 536)
Exacerbation in one year follow-up, Median (IQR)	0 (1)
Exacerbation in one year follow-up *n* (%)	
Yes	193 (36.0)
No	343 (64.0)
Frequent exacerbation in one year follow-up, *n* (%)	
Yes	86 (16.0)
No	450 (84.0)
Severe exacerbation in one year follow-up, *n* (%)	
Yes	117 (21.8)
No	419 (78.2)

### Correlation of baseline CAT score and change in CAT with future exacerbations

**Figure 2.**
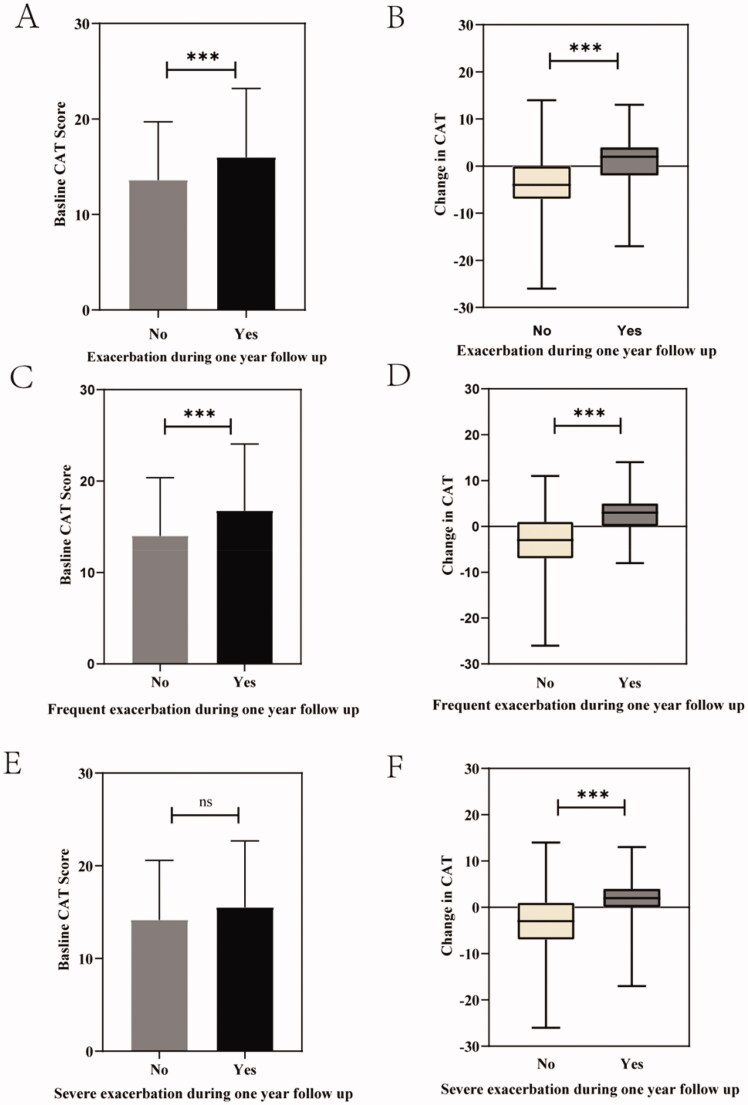
Baseline CAT score and change in CAT in the exacerbation of the follow-up year. (A) Baseline CAT score in patients with exacerbation and without acute exacerbation in 18 months follow up; (B) Change in CAT in patients with exacerbation and without exacerbation in 18 months follow up; (C) Baseline CAT score in patients with frequent exacerbation and without frequent exacerbation in 18 months follow up; (D) Change in CAT in patients with frequent exacerbation and without frequent exacerbation in 18 months follow up; (E) Baseline CAT score in patients with severe exacerbation and without severe exacerbation in 18 months follow up; (F) Change in CAT score in patients with severe exacerbation and without severe exacerbation in 18 months follow up. Data were compared between groups using t-tests or the Kruskal-Wallis *H* test. ns indicates *p*-values ≥ .05, *** indicates *p*-values < .001. Abbreviations: CAT, COPD Assessment Test; Change in CAT, CAT score changing between baseline and the 6-month follow-up.

The study compared baseline CAT scores and changes in CAT between patients with different numbers and severity of exacerbations during one-year follow-up. As shown in Figure 2, the mean baseline CAT score of patients with exacerbation was higher than that of patients without exacerbation (*p* < .001). Additionally, it showed a different median change in CAT between the two groups; the CAT score of patients with exacerbation during one-year follow-up from 0^th^ to 12^th^ month had deteriorated at the 6-month follow-up compared to baseline but had decreased in the patients without future exacerbation [2 (6) versus −4 (7), *p* < .001]. Compared to patients without frequent exacerbations during the follow-up from 0^th^ to 12^th^ month, patients with frequent exacerbations had a higher mean baseline CAT (*p* < .001) and a worse change in CAT (*p* < .001) at the 6-month follow-up. However, patients with severe exacerbations showed no significant difference in baseline CAT (*p* > .05) compared to patients with non-severe exacerbations, but they did show a greater CAT score deterioration at the 6-month visit (*p* < .001).

**Figure 3.**
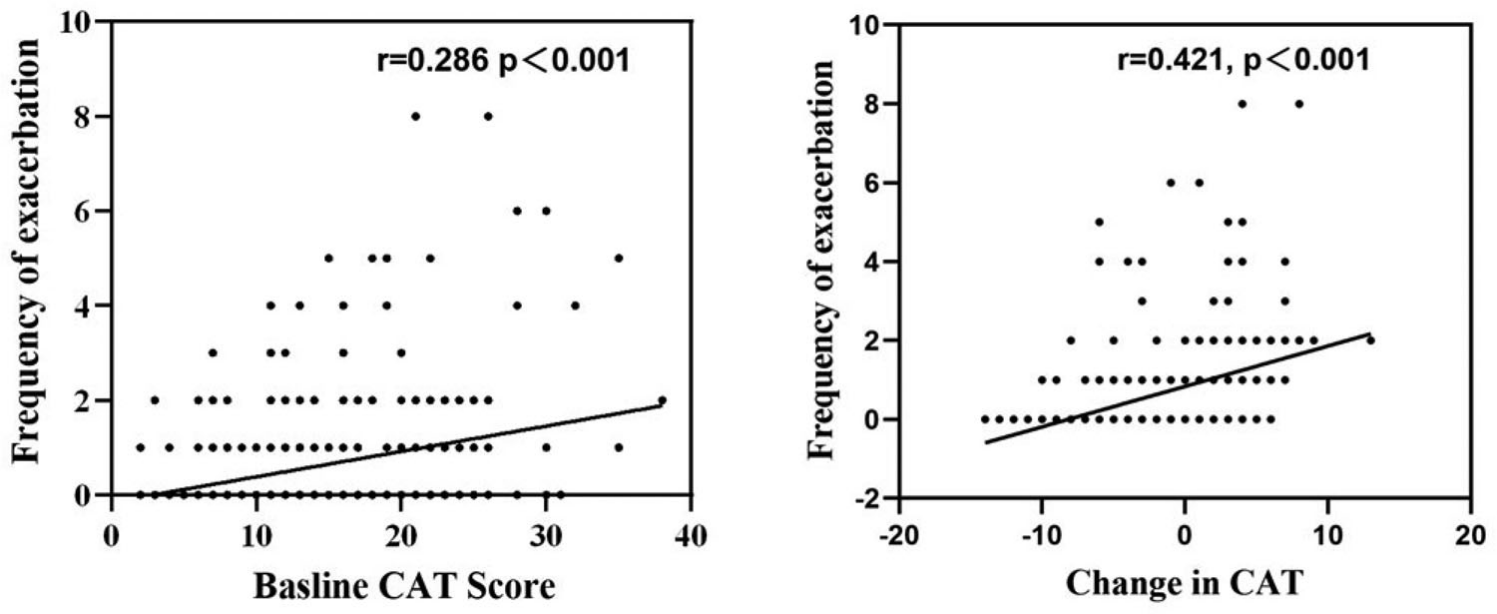
The association between the Baseline CAT score and Change in CAT with the frequency of acute exacerbation. (A) The association between the Baseline value of CAT and the frequency of exacerbation; (B) The association between Change in CAT and the frequency of exacerbation. Correlation between continuous variables was evaluated using Spearman's rank correlation coefficient. Abbreviations: CAT, COPD Assessment Test; Change in CAT, CAT score changing between baseline and the 6-month follow-up.

As shown in Figure 3, both show a positive correlation with the frequency of future exacerbation. The correlation between baseline CAT score and frequency of exacerbations during the one year of follow-up is a week (*r* = 0.286, *p* < .001), the correlation between change in CAT score and frequency of exacerbation during one-year follow up was moderate (*r* = 0.421, *p* < .001).

By univariate Cox regression analysis, we accessed the risk factors associated with the occurrence of at least one exacerbation in the one-year follow-up. The meaningful predictive factors included FEV1% predicted, baseline CAT score, mMRC score, CCQ score, exacerbations in the past year, GOLD Grade, Change in CAT and MPR (Table 4). These variables were included in the multivariate Cox regression model. Finally, baseline CAT score [hazard ratio (HR): 1.082; 95% confidence interval (CI): 1.0045–1.120; *p* < .001]; Change in CAT (HR: 1.203; CI: 1.160–1.247; *p* < .001) and exacerbation in the previous year (HR: 1.427; CI: 1.144–1.778; *p* < .001) were the best predictor of future exacerbation (Table 5).

**Table 4.** Univariate Cox regression analysis of factors predicting exacerbation during the one-year follow-up.

Factors	HR	95% CI	*P*-value
Age	1.008	0.991–1.021	.348
Sex (Male versus Female)	1.099	0.862–1.400	.477
Smoking status	1.018	0.828–1.323	.924
FEV1	1.034	0.974–1.098	.272
FEV1 (% predicted)	0.991	0.984–0.999	.038
FEV1/FVC			.486
Baseline CAT score	1.046	1.024–1.067	.000
mMRC (0–1 versus 2–4)	1.602	1.158–2.215	.004
CCQ	1.019	1.003–1.035	.022
Exacerbations in the past year (Yes versus No)	1.743	1.468–2.070	.000
GOLD	0.995	0.983–1.008	
I	Reference		
II	1.493	0.759–2.938	.246
III	2.086	1.064–4.088	.032
IV	2.565	1.246–5.281	.011
Therapy	1.109	0.995–1.236	.061
Change in CAT	1.150	1.117–1.184	.000
MPR (Adherence versus No adherence)	0.689	0.577–0.901	.022

Abbreviations: CAT, COPD Assessment Test; Change in CAT, CAT score changing between baseline and the 6-month follow-up; CCQ, Clinical COPD Questionnaire; FEV1, Forced Expiratory Volume in one second; FVC, Forced Vital Capacity; GOLD, Global Initiative for Chronic Obstructive Lung Disease; mMRC, modified medical research council dyspnoea scale; MPR, medication possession ratio.

**Table 5.** Multivariate Cox regression analysis of factors predicting exacerbation during the one-year follow-up.

Factors	HR	95% CI	*P*-value
FEV1 (% predicted)	0.994	0.978–1.011	.506
Baseline CAT score	1.082	1.045–1.120	.000
mMRC (0–1 versus 2–4)	1.218	0.793–1.875	.247
CCQ	1.008	0.985–1.003	.461
Exacerbations in the past year (Yes versus No)	1.427	1.144–1.778	.000
GOLD grade			
I	Reference		
II	1.174	0.532–2.590	.691
III	1.076	0.410–2.826	.882
IV	1.176	0.388–3.563	.775
Change in CAT	1.203	1.160–1.247	.000
MPR (Adherence versus No adherence)	0.891	0.755–1.089	.347

Notes: FEV1% predicted, Baseline CAT score, mMRC score,CCQ score, exacerbations in the past year, GOLD Grade, Change in CAT and MPR were included as the variables in the multivariate Cox regression model.

Abbreviations: CAT, COPD Assessment Test; Change in CAT, CAT score changing between baseline and the 6-month follow-up; CCQ, Clinical COPD Questionnaire; FEV1, Forced Expiratory Volume in one second; GOLD, Global Initiative for Chronic Obstructive Lung Disease; mMRC, modified medical research council dyspnoea scale; MPR, medication possession ratio.

### Comparation of the predictive value of CAT baseline value and change in CAT for future exacerbations

**Figure 4.**
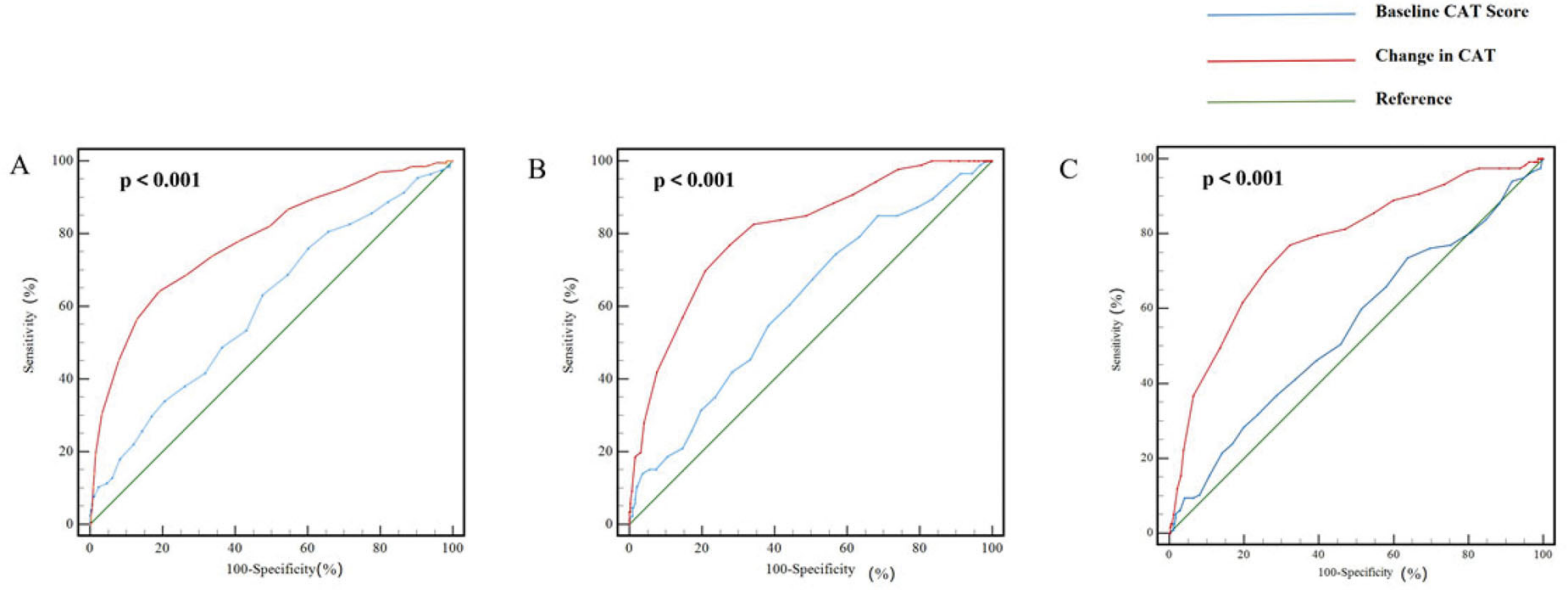
Predictive value of Baseling CAT score and Change in CAT for future exacerbation. (A) Predictive value of Baseling CAT score and Change in CAT for the occurrence of exacerbation; (B) Predictive value of Baseling CAT score and Change in CAT for the frequent exacerbation; (C) Predictive value of Baseling CAT score and Change in CAT for the severe exacerbation. Comparing area ounder the ROC curve (AUC) of baseline AUC of CAT score and Change in CAT for acute exacerbation of COPD patients were compared by z statistic. AUC of Baseline CAT score and Change in CAT for predicting the occurrence of exacerbation is 0.609 and 0.789 (*p* < .001), the sensitivity is 75.9% and 64.1%, the specificity is 60.1% and 70.9%; AUC of Baseline CAT score and Change in CAT for predicting frequent exacerbation is 0.611 and 0.805 (*p* < .001), the sensitivity is 74.4% and 76.7%, the specificity is 43.1% and 73.4%; AUC of Baseline CAT score and Change in CAT for predicting severe exacerbation is 0.550 and 0.771 (*p* < .001), the sensitivity is 73.5% and 76.9%, the specificity is 36.3% and 67.8%. Abbreviations: CAT, COPD Assessment Test; Change in CAT, CAT score changing between baseline and the 6-month follow-up; ROC, Receiver operating characteristic.

ROC curves showed that change in CAT had a better predictive capacity for the occurrence of exacerbation than baseline CAT (0.789 versus 0.609, *p* < .001). The sensitivity for identifying exacerbation of change in CAT and baseline CAT score was 64.1% and 75.9%, and the specificity was 70.9% and 60.1%, respectively. In addition, change in CAT had a better predictive value for frequent exacerbation compared with baseline CAT score; the area under the ROC curve (AUC) was 0.805 and 0.611 (*p* < .001). Additionally, change in CAT also showed a better predictive capacity for severe exacerbation than baseline CAT; the AUC was 0.771 and 0.550 (*p* < .001) (Figure 4).

### Comparison of predictive value of change in CAT and exacerbation in the past year for future exacerbations

**Figure 5.**
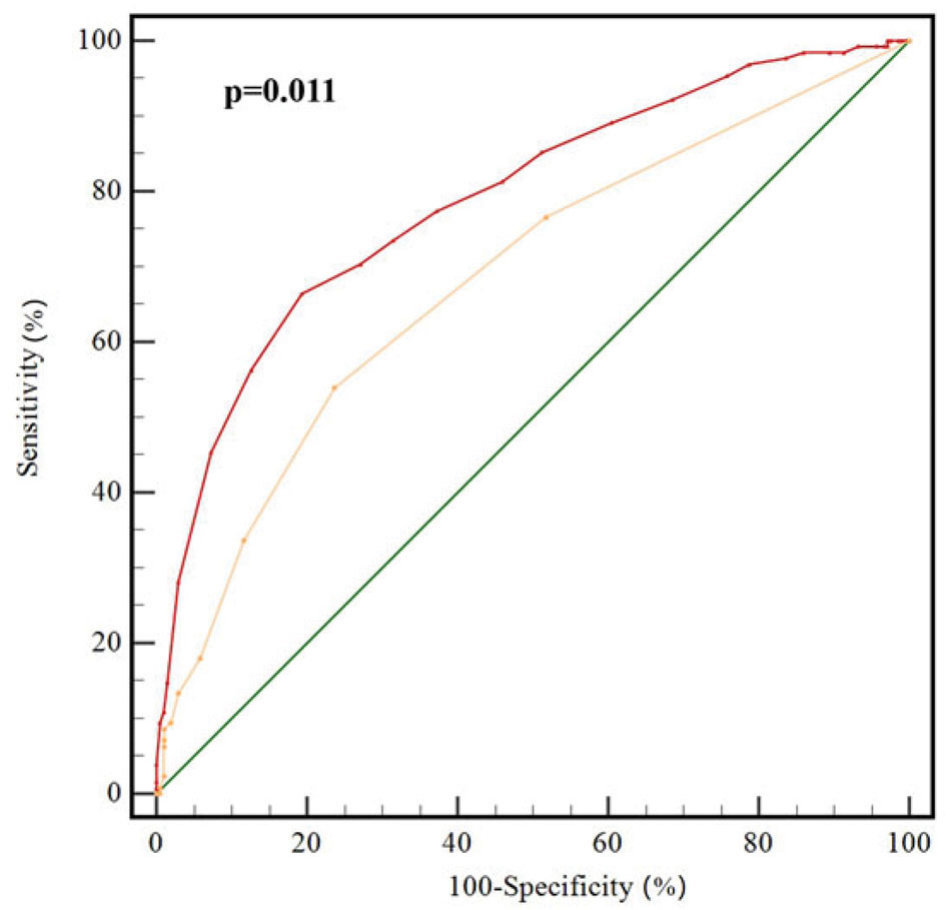
Predictive value of change in CAT and acute exacerbations in the past year for future exacerbation. AUC of Change in CAT and exacerbation in the past year for acute exacerbation of COPD patients was compared by z statistic. AUC of Baseline CAT and Change in CAT for predicting the occurrence of exacerbation is 0.789 and 0.689 (*p* = .011), the sensitivity is 75.3% and 78.1%, the specificity is 35.2% and 72.7%. Abbreviations: CAT, COPD Assessment Test; Change in CAT, CAT score changing between baseline and the 6-month follow-up; ROC, Receiver operating characteristic.

We compared the value of change in CAT and exacerbations in the past year for predicting the occurrence of future exacerbation. As shown by Figure 5, ROC curves showed that change in CAT had a slightly better predictive value for the occurrence of exacerbation than exacerbation in the past year (AUC = 0.789 versus 0.689, *p* = .011). The optimal cut-off for change in CAT value is 2. The patients were divided into groups with change in CAT ≥ 2 and change in CAT < 2. Comparing the exacerbations in the previous year, there was no significant difference between the two groups (Table 6).

**Table 6.** The clinical characteristics in the difference Change in the CAT group.

Variables	ΔCAT ≥ 2	ΔCAT<2	*P*-value
Age (years)^a^	63.8 (8.3)	64.1 (8.4)	.774
Sex^b^			.351
Male	137 (89.0)	340 (89.0)	.899
Education			.607
Primary school^b^	51 (33.1)	156 (40.9)	
Junior^b^	66 (42.9)	139 (36.4)	
High school^b^	25 (16.2)	59 (15.4)	
University^b^	12 (7.8)	28 (7.3)	
Baseline CAT score^a^	12.9 (6.0)	14.6 (6.6)	.022
mMRC^c^	2 (2)	2 (2)	.113
0–1^b^	40 (26)	141 (36.9)	.015
2–4^b^	114 (74)	241 (63.1)	
CCQ^a^	19.1 (9.4)	18.4 (8.7)	.684
FEV1 (% predicted)^c^	49.1 (27.8)	51.3 (29.2)	.090
FEV1/FVC^c^	44.1 (19.4)	45.3 (20.6)	.523
Exacerbations in the past year^c^	1 (2)	1 (2)	.176
Exacerbations in the past year^b^			.113
0	58 (37.7)	167 (43.7)	
≥1	96 (62.3)	215 (56.3)	
Frequent exacerbation in the past year^b^			.587
Yes	38 (33.0)	128 (30.4)	
No	77 (67.0)	293 (69.6)	
Gold grade^b^			.042
I	7 (4.5)	41 (10.7)	
II	63 (40.9)	150 (39.3)	
III	70 (45.5)	136 (35.6)	
IV	14 (9.1)	55 (14.4)	
Therapies^b^			.809
LAMA	44 (28.6)	120 (32.0)	
ICS + LABA	17 (11.0)	35 (9.3)	
LABA + LAMA	10 (6.5)	28 (5.3)	
ICS + LABA + LAMA	82 (53.2)	192 (51.2)	
Others	1 (0.7)	7 (1.8))	
MPR in the 6 month^b^			.001
Adherencee	79 (51.3)	258 (67.5)	
No adherence	75 (48.7)	124 (32.5)	

^a^Mean（SD); ^b^Counts with percentage are indicated; ^c^Median (IQR).

Abbreviations: COPD, Chronic Obstructive Pulmonary Diseas; CAT, COPD Assessment Test; CCQ, Clinical COPD Questionnaire; FEV1, Forced Expiratory Volume in one second; FVC, Forced Vital Capacity; GOLD, Global Initiative for Chronic Obstructive Lung Disease. ICS, inhaled corticosteroids; IQR, interquartile range; LABA, long-acting β-2-agonist; LAMA, long-acting muscarinic antagonist; mMRC, modified medical research council dyspnoea scale; MPR: medication possession ratio.

### The clinical characteristic associated with change in CAT

The change in CAT was divided into two groups according to the cut-off value. compared with patients with a change in CAT ≤ 2, the Baseline CAT score was slightly lower, more patients with mMRC ≥ 1, fewer patients with GOLD I, more patients with GOLD III, and more patients with poor medication adherence in patients with change in CAT ≥ 2 (Table 6). After adjusting for sex, age, baseline CAT score, GOLD stage and MPR, the multivariate logistic regression model showed that patients in GOLD stage II (OR: 2.805, 95% CI: 1.163–6.765 *p* = .022) and (OR: 4.342, 95% CI: 1.764–10.685 *p* = .001) III were associated with more than 2 scores worsening in the change in CAT comparing with GOLD stage I, and patients with poor adherence (OR: 1.733, 95% CI: 1.202–2.497 *p* = .003) had a higher incidence of more than 2 scores worsening in a change in CAT (Table 7).

**Table 7.** Factors associated with a different changes in CAT in the multivariate model.

Factors	OR	95% CI	*P*-value
GOLD			.011
I	Reference		
II	2.805	1.163–6.765	.022
III	4.342	1.764–10.685	.001
IV	2.475	0.901–6.796	.079
MPR	1.733	1.202–2.497	.003

Notes: Baseline CAT score, mMRC score, GOLD Grade, Change in CAT and MPR were included as the variables in the multivariate logistic regression model.

Abbreviations: GOLD, Global Initiative for Chronic Obstructive Lung Disease; MPR, medication possession ratio.

## Discussion

To the best of our knowledge, this is the first prospective, observational study to compare the predictive value of the baseline CAT score and the change in CAT for future exacerbation. COPD exacerbation is one of the important events in the disease progression of COPD patients. A study showed that severe exacerbation is an independent risk factor for death [19]. Therefore, it is necessary to assess the risk of exacerbations. Recent research has shown that a history of exacerbations in the previous year was associated with a higher risk of future exacerbation [7,20]. This result is consistent with our research, in which patients with exacerbations in the past year were more likely to experience exacerbations during the one-year follow-up compared to those without a history of exacerbation. Besides, the study showed that history of exacerbation was one of the independent risk factors for future exacerbation on the multivariate Cox model.

The research showed that patients who experience future exacerbation have a higher baseline CAT score than patients who do not, and there are similar phenomena seen in patients with frequent exacerbations and infrequent exacerbations. This result is in line with previous research [10,21], in which patients with a higher CAT score were at higher risk of exacerbation.

The study showed that the CAT scores at 6-month visits deteriorated from the baseline in patients with exacerbation during the one-year visit compared to patients without exacerbation. This phenomenon was also observed in patients with frequent and severe exacerbation. This is consistent with previous research showing that CID-C based on CAT deterioration was associated with the risk of future exacerbation [15]. This reflects that the occurrence of short-term CAT deterioration may be associated with future exacerbations. In addition, studies of Claus et al. [22] have regarded a short-term deterioration in CAT greater than 2 points as a part of CID of COPD patients to assess changes in disease status over 20 weeks. The research found that not only history of exacerbation and CAT score, but also change in CAT were also one of the independent risk factors for future exacerbation on multivariate Cox model, this is a new discovery that is different from CID to predict future exacerbation.

Moreover, this study found that baseline CAT value has a positive correlation and predictive value for future exacerbation risk. This is consistent with the previous results, in which a CAT score over 15 is a good model for predicting exacerbations in the future, with an AUC of 0.610 [11]. In addition, the study showed that change in CAT was also positively correlated with the frequency of exacerbation in the one-year follow-up. Change in CAT can not only predict the possibility of future exacerbation and frequent exacerbation, but also the risk of severe exacerbation. Therefore, these results showed that in addition to the health status at a time in point, the changes in health status may be also an important basis for predicting future exacerbations. This is consistent with the study by Naya et al. [14], in which CID containing SGRQ for assessment of a healthy quality of life over 6 months was also associated with future exacerbation. The difference is that our study is the first to analyse the relationship between changes in CAT scores and future exacerbations separately. Also, the change in CAT value had a higher *r* value and a better predictive value for future exacerbation than the baseline CAT score in the study, which represents a novel discovery.

History of exacerbations has been widely used to assess future exacerbations, but it relies on the subjective recollection of patients, leading to misdiagnosis and missed diagnosis. Therefore, a more objective and effective basis is needed for clinical treatment. The study analysed the predictive value of exacerbation in the past year, demonstrating an AUC of 0.698 (*p* < .001). It is consistent with the previous research, in which the exacerbation in the past year had predictive capacity for future exacerbation, with an AUC of 0.609 [21]. Further analysis of our research found that the change in CAT has better predictive value for future exacerbations than exacerbation in the previous year. History of exacerbations is an important basis for evaluation of the disease and treatment [23]. However, short-term activity trajectories can better reflect the current status of the disease. CID can be used to assess the early deterioration of the COPD trajectory and can provide useful information on how to maintain patient stability [12,24]. At present, more and more research is being devoted to drugs for the prevention and treatment of CID, so as to prevent disease deterioration [25–27].

The study also analysed the clinical feature in the different changes in the CAT group, it indicated GOLD stage II, III and medication adherence were significantly associated with a change in CAT in logistic regression analysis. Previous studies have reported that patients with high GOLD stage were more likely to experience disease exacerbations [28], and CAT scores increased when they experienced exacerbations [29]. In this study, only GOLD II and III were associated with a change in CAT in this study, GOLD grade IV was not a related factor for the deterioration of CAT, which deserves further study. Moradkhani B et al [30] showed that patients with high quality of life were more adherent to their medications, therefore patients with poor adherence would develop deterioration of CAT scores in short term.

CAT is a common clinical test used to assess the health status of COPD patients. As a part of CID, change in CAT can be more objective and simple to reflect short-term disease status compared with the evaluation three parts in CID. Therefore, change in CAT can be observed as health status changes to provide clinicians with a more objective and reliable clinical measure to predict the risk of disease aggravation.

There are some limitations of this study. Firstly, we established a good predictive value of changes in CAT in one cohort but lacked another cohort to verify our model. Secondly, although this study also shows the change in CAT has better predictive value for future exacerbation than the history of exacerbation, it does not compare the value of a series of measurements and a single current measurement associated with the trajectory of disease to predict the risk of exacerbation. Since our study uses an ongoing prospective design, further studies can be carried out.

## Conclusion

In summary, our results revealed that a higher baseline CAT score and worse change in CAT in the short term were associated with a higher risk of future exacerbation. The correlation between baseline CAT score and future exacerbation was weak, however, the correlation between change in CAT and future exacerbation was moderate. Short-term change in CAT had a better predictive value for future exacerbations of COPD than baseline CAT and exacerbation in the previous year.

## Supplementary Material

Supplemental Material

## Data Availability

All publications discussed in the manuscript are available from the corresponding author on request.

## Abbreviations

BMI: Body Mass Index
COPD: Chronic Obstructive Pulmonary Disease
CAT: COPD Assessment Test
CID: clinically important deterioration
FEV1: Forced Expiratory Volume in one second
FVC: Forced Vital Capacity
GOLD: Global Initiative for Chronic Obstructive Lung Disease
ICS: inhaled corticosteroids
IQR: interquartile range
LABA: long-acting β-2-agonist
LAMA: long-acting muscarinic antagonist
MCID: minimal clinically important difference
MPR: medication possession ratio
mMRC: modified Medical Research Council
ROC: The receiver operating characteristic
SGRQ: St George's Respiratory Questionnaire.
