# Supplementary material for: Comparation of predictive value of CAT and change in CAT in the short term for future exacerbation of chronic obstructive pulmonary disease
Source: Ann Med. 2022 Mar 26;54(1):875–85. doi: 10.1080/07853890.2022.2055134 (PMC8959516; doi:10.1080/07853890.2022.2055134)
Supplement: Supplemental Material [file IANN_A_2055134_SM7402.docx]

**Table S1. Exacerbation during the one year follow up according to exacerbation in the previous year**

| **Variables** | **Exacerbation in the previous year (n=331)** | **No exacerbation in the previous year (n=225)** | **P-value** |
| --- | --- | --- | --- |
| Exacerbation in one year follow-up,  Median (IQR) | 0 (1) | 0 (0) | <0.001 |
| Exacerbation in one year follow-up, n(%) |  |  | <0.001 |
| Yes | 149 (47.9) | 46 (20.4) |  |
| No | 162 (52.1) | 179 (79.6) |  |
| Frequent exacerbation in one year follow-up,n(%) |  |  | <0.001 |
| Yes | 76 (24.4) | 14 (6.2) |  |
| No | 235 (75.6) | 211 (93.8) |  |
| Severe exacerbation in one year follow-up,n(%) |  |  | <0.001 |
| Yes | 89 (28.6) | 27 (12.0) |  |
| No | 222 (71.4) | 198 (88.0) |  |

**Abbreviations:** CAT, COPD Assessment Test; Change in CAT, CAT score changing between baseline and the 6-month follow-up; ROC, Receiver operating characteristic.
